# Psychological disturbances and their association with sleep disturbances in patients admitted for cardiovascular diseases

**DOI:** 10.1371/journal.pone.0244484

**Published:** 2021-01-04

**Authors:** Risa Matsuda, Takashi Kohno, Shun Kohsaka, Yasuyuki Shiraishi, Yoshinori Katsumata, Kentaro Hayashida, Shinsuke Yuasa, Seiji Takatsuki, Keiichi Fukuda

**Affiliations:** 1 Division of Cardiology, Department of Medicine, Keio University School of Medicine, Tokyo, Japan; 2 Department of Cardiovascular Medicine, Kyorin University School of Medicine, Tokyo, Japan; 3 Institute for Integrated Sports Medicine, Keio University School of Medicine, Tokyo, Japan; University of Illinois at Chicago College of Medicine, UNITED STATES

## Abstract

**Background:**

Depression and anxiety are common mental health problems that are strongly associated with sleep disturbances, according to community-based researches. However, this association has not been investigated among patients admitted for cardiovascular diseases (CVDs). We examined the prevalence of depression and anxiety in inpatients with various CVDs and their association with sleep disturbances.

**Materials and methods:**

This cross-sectional study included 1294 patients hospitalized for CVDs in a Japanese university hospital were evaluated for their mental status using the Hospital Anxiety and Depression Scale (HADS), for sleep-disordered breathing (SDB) using pulse oximetry, and for sleep quality using the Pittsburgh Sleep Quality Index (PSQI).

**Results:**

Patient characteristics were as below: mean age, 63.9±14.7 years; 25.7% female. Overall, 18.9% had depression (HADS-depression≥8) and 17.1% had anxiety (HADS-anxiety≥8). The presence of depression was associated with female sex, older age, higher plasma brain natriuretic peptide level, lower estimated glomerular filtration rate, and the prevalence of heart failure. Overall, 46.5% patients were categorized as having a poor sleep quality (PSQI>5), and 28.5% patients had SDB (3% oxygen desaturation index>15). Although depression and anxiety were not associated with SDB, they were independently associated with poor sleep quality (OR = 3.09, 95% CI 2.19–4.36; OR = 3.93, 95% CI 2.71–5.69, respectively).

**Conclusions:**

Depression and anxiety were not uncommon in patients with CVDs. Poor sleep quality could be an important risk factor linked to psychological disturbances.

## Introduction

Depression and anxiety are frequently encountered mental health problems in association with increased morbidity and mortality, greater financial burden, and reduced quality of life [1, 2]. Accumulating evidence based on community-based researches has highlighted the importance of sleep health for mental health [3–7]. Sleep-disordered breathing (SDB), the most commonly investigated among sleep disturbances in cardiovascular diseases (CVDs) [3], was associated with depression in a community-based cohort of middle-aged and older adults [6]. According to cohort studies based on the general population, poor quality of sleep was independently associated with the development of anxiety and depression [8, 9].

Current international guidelines recommend assessment for depression and anxiety as comorbidities as well as risk factors of CVDs using standardized questionnaires [10, 11]. However, both conditions are underdetected and consequently undertreated in the real-world cardiology setting, particularly in inpatients who required acute care [12]. Furthermore, the association between psychological disturbances and SDB was determined by population-based cohort studies [4–7]. Thus, the prevalence of psychological disturbances and their association with sleep disturbances remain unknown among CVD inpatients.

The aim of this study was to evaluate the prevalence of depression and anxiety and their relationship with sleep disturbances, including SDB and poor sleep quality, in hospitalized patients with CVDs.

## Materials and methods

### Study population and design

This study was a single-center, cross-sectional study. A total of 2110 consecutive patients who were admitted for various CVDs as the primary diagnosis and who had undergone pulse oximetry during hospitalization for the screening of SDB in the general ward of the Cardiology Department of Keio University Hospital from September 2013 to December 2015, were included in the study. The patients who refused to participate in the study were excluded from the study. Altogether, 217 patients from whom the completed questionnaire data could not be obtained (declined to answer the questionnaires or had conditions that made it arduous to complete a self-administered written questionnaire [e.g., dementia, delirium, unconsciousness]) and 549 patients who had submitted an incomplete questionnaire, were excluded from the study. The dataset for this analysis comprised of individual patients at one time point. In cases where a patient was admitted to our hospital more than once and underwent additional screenings during the study period, these subsequent assessments were excluded (n = 50). Finally, 1294 patients were included in this analysis. This study’s protocol conformed to the ethical guidelines of the 1975 Declaration of Helsinki as reflected in a priori approval by the Institutional Review Board of Keio University School of Medicine. The Institutional Review Board in our institution approved the procedure of obtaining verbal consent from the individuals and accordingly, verbal consent was obtained from all patients prior to their enrollment in the study. A separate filled in questionnaire sheet was also obtained from the patients.

### Data collection

Clinical data were extracted from the patient’s hospital chart, including sex, age, body mass index (BMI), lifestyle (job status, smoking, alcohol intake, and living conditions), cardiovascular risk factors, laboratory data (albumin, brain natriuretic peptide [BNP], C-reactive protein [CRP], estimated glomerular filtration rate [eGFR], and hemoglobin A1c [HbA1c]), and history and admission diagnoses of CVD.

Anxiety and depression were assessed using the Hospital Anxiety and Depression Scale (HADS) [13], which is a widely used, self-reported questionnaire developed to assess the anxiety and depression levels in population studies, primary care, and hospital settings [14]. It consists of 14-items on a 4-point Likert scale (range 0 [not present] to 3 [maximally present]), and is designed to evaluate anxiety (HADS-anxiety) and depression (HADS-depression), respectively (7 items for each subscore), resulting in a potential range of subscores from 0 to 21. We chose the HADS in this study after careful discussion because the somatic symptoms regarding sleep quality (e.g. insomnia and fatigue) were not included in this questionnaire. The majority of the symptoms in the HADS‐anxiety are indices of generalized anxiety, with only one item related to panic. The HADS-depression primarily comprises symptoms of anhedonia.

Overnight pulse oximetry (PULSOX-Me300, Teijin Co., Ltd, Tokyo, Japan) was performed to assess the severity of SDB in all patients. As we reported previously [15], arterial blood oxyhemoglobin saturation was recorded using a finger probe at a 1-Hz sampling frequency and 5-second average time, and these recordings were analyzed using the software supplied with the equipment (DS-Me, Teijin Co., Ltd, Tokyo, Japan). As an indicator of SDB severity, we chose the 3% oxygen desaturation index (3% ODI), which represents the number of events of 3% desaturation per hour. The validity of the pulse oximetry was reported based on its synchronous overnight recording with polysomnography, and its sensitivity and specificity were 85% and 100%, respectively, for detecting an apnea hypopnea index of ≥ 20 determined by polysomnography using a cut-off threshold of 3% ODI = 15 [15–17]. The Japanese version of the Pittsburgh Sleep Quality Index (PSQI) was used to assess the sleep quality [18]. The PSQI consists of 19 self-rated questionnaires, which are grouped into 7 components, including sleep duration, sleep disturbances, sleep latency, daytime dysfunction due to sleepiness, habitual sleep efficiency, overall sleep quality, and sleeping medication use. Each component yields a score ranging from 0 to 3, with 3 indicating the greatest dysfunction. Patients completed these questionnaires during their index hospitalization, most of them within a few days after hospital admission. The patients who initially required intensive care received these questionnaires before discharge.

### Definitions and outcomes

Depression and anxiety were defined as HADS-depression score ≥8 and HADS-anxiety score ≥8, respectively, as originally recommended for identifying clinically significant depression and anxiety, providing the optimal balance between sensitivity and specificity for identifying cases [13, 14]. SDB and poor sleep quality were defined as 3% ODI ≥ 15 and PSQI > 5, respectively based on commonly used clinical thresholds [17, 19].

### Statistical analysis

Continuous variables were expressed as mean ± standard deviation for normally distributed variables and median (inter-quartile range) for non-normally distributed variables (HADS-anxiety, HADS-depression, albumin, BNP, and HbA1c). Categorical variables were expressed as absolute values (percentages). Clinical characteristics, laboratory data, pulse oximetry data, and questionnaires were compared between patients with depression or anxiety and those without. Student’s *t* test and Mann-Whitney *U* test were used to compare the normally or non-normally distributed variables, and chi-square test was used to compare the categorical variables. Cronbach’s α was calculated to assess the internal consistency of both the HADS anxiety and depression scores and the PSQI total score. Multiple logistic regression models were used to evaluate the independent determinants of depression or anxiety by adjusting for age, sex, obesity, living status, poor sleep quality (PSQI > 5), SDB, cardiovascular comorbidities (heart failure [HF], coronary artery disease [CAD], and atrial fibrillation [AF]), cardiovascular risk factors (hypertension, diabetes, and dyslipidemia), and laboratory data (albumin, CRP, and eGFR). Further, a multiple logistic regression model was also used to evaluate the association between psychological disturbances (anxiety alone, depression alone, and both anxiety and depression) and sleep disorders. Multicollinearity was assessed before the multiple logistic regression analyses; the factors indicating serious multicollinearity were accordingly eliminated from the model. *P* values less than 0.05 were considered statistically significant. The analyses were performed using IBM SPSS 24.0 for Windows (SPSS Inc., Chicago, IL, USA).

## Results

### Sample characteristics

Of the 1294 enrolled patients (mean age; 63.9±14.7 years; 333 [25.7%] female), 401 (31.0%) had CAD as an admission diagnosis (acute coronary syndrome [ACS] n = 71, non-ACS n = 330), 90 (7.0%) had HF, 521 (40.3%) had arrhythmias, 145 (11.2%) had valvular heart diseases (VHDs), and 137 (10.6%) had other conditions. The subscores of HADS-depression and anxiety were 4.0 [2.0–7.0] and 4.0 [2.0–6.0], respectively. HADS-D and HADS-A subscores were correlated with each other (r = 0.41, p<0.001).

### Clinical characteristics of patients by psychological disturbances

**Fig 1:**
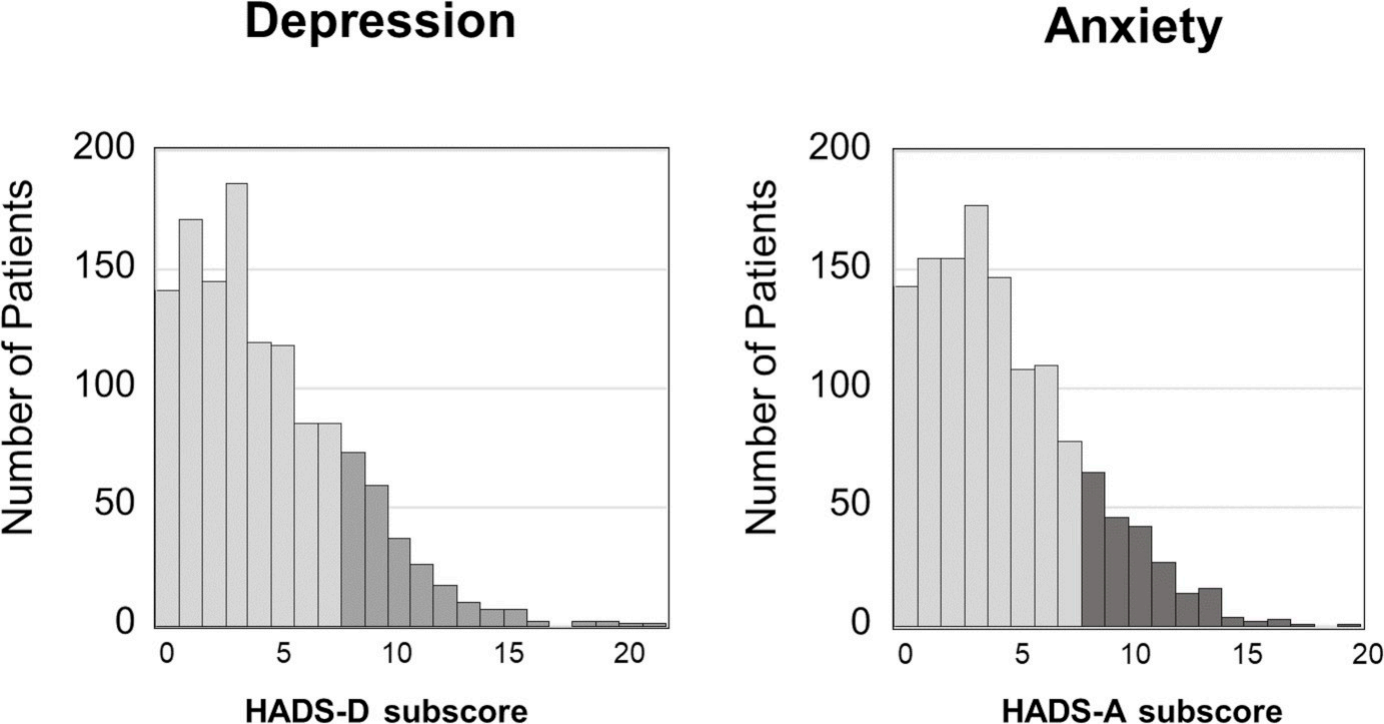
Distribution of the hospital anxiety and depression scale. HADS-A, Hospital Anxiety and Depression Scale subscore for anxiety. HADS-D, Hospital Anxiety and Depression Scale subscore for depression.

Of 1294 patients, 244 patients (18.9%) were categorized as having depression symptoms (HADS-depression≥8) and 221 patients (17.1%) were categorized as having anxiety symptoms (HADS-anxiety≥8) (Fig 1). The baseline clinical characteristics of the study population are shown in Table 1. The presence of depression was associated with female sex, older age, higher plasma BNP level, lower eGFR, and the prevalence of HF. No significant differences in the patients’ characteristics and laboratory data were observed between patients with different anxiety status. The internal consistency reliability measured with Cronbach’s α was 0.78 for HADS-anxiety and 0.76 for HADS-depression. The PSQI global score had an α = 0.65, a value suggesting moderate consistency. Cronbach’s α increased by 0.014 (from 0.65 to 0.67) on removing the PSQI component associated with sleep medication usage.

**Table 1:** Clinical characteristics of patients by depression or anxiety status.

		Depression	Non-Depression		Anxiety	Non-Anxiety	
Variables	Total	HADS-D≥8	HADS-D<8	p value	HADS-A≥8	HADS-A<8	p value
	n = 1294	n = 244	n = 1050		n = 221	n = 1073	
Female n (%)	333 (25.7)	82 (33.6)	251 (23.9)	0.002	66 (29.9)	267 (24.9)	0.123
Age (y)	63.9±14.7	66.6±15.0	63.3±14.6	0.002	63.3±15.8	64.0±14.5	0.533
Body mass index (kg/m^2^)	23.9±3.7	23.5±4.1	24.0±3.6	0.084	23.9±4.1	23.9±3.7	0.972
Medical history n (%)							
CAD	505 (39.5)	94 (39.8)	411 (39.5)	0.921	75 (34.4)	430 (40.6)	0.088
Heart Failure	228 (17.8)	61 (25.7)	167 (16.0)	<0.001	47 (21.7)	181 (17.1)	0.107
Atrial fibrillation	472 (37.3)	81 (34.9)	391 (37.9)	0.397	81 (37.5)	391 (37.3)	0.958
Hypertension	700 (54.4)	133 (55.2)	567 (54.3)	0.794	131 (59.5)	569 (53.4)	0.094
Diabetes mellitus	280 (21.8)	58 (24.1)	222 (21.3)	0.346	45 (20.5)	235 (22.1)	0.594
Dyslipidemia	573 (45.4)	113 (48.9)	460 (44.6)	0.235	94 (43.9)	479 (45.7)	0.633
Lifestyle n (%)							
Smoking	130 (10.1)	28 (11.6)	102 (9.8)	0.413	24 (11.0)	106 (10.0)	0.641
Alcohol	716 (55.9)	107 (44.2)	609 (58.7)	<0.001	110 (50.0)	606 (57.2)	0.051
Living alone	188 (15.4)	38 (16.9)	150 (15.0)	0.481	34 (16.7)	154 (15.1)	0.571
Employed	641 (58.4)	98 (46.7)	543 (61.2)	<0.001	105 (56.8)	536 (58.8)	0.612
HADS-Anxiety	4.0 [2.0–6.0]	7.0 [5.0–10.0]	3.0 [1.0–5.0]	<0.001	9.0 [8.0–11.0]	3.0 [1.0–5.0]	<0.001
HADS-Depression	4.0 [2.0–7.0]	9.0 [8.0–11.0]	3.0 [1.0–5.0]	<0.001	8.0 [6.0–10.0]	3.0 [1.0–5.0]	<0.001
Laboratory data							
CRP (mg/dL)	0.06 [0.02–0.20]	0.06 [0.02–0.25]	0.06 [0.02–0.19]	0.746	0.07 [0.02–0.28]	0.06 [0.02–0.19]	0.316
Albumin (g/dL)	4.2 [3.8–4.4]	4.1 [3.8–4.3]	4.2 [3.8–4.4]	0.051	4.1 [3.8–4.4]	4.2 [3.8–4.4]	0.547
BNP (pg/mL)	57 [22–144]	75 [24–194]	53 [21–140]	0.016	60 [19–138]	56 [22–146]	0.809
eGFR (ml/min/1.73m^2^)	62±18	59±20	63±18	0.008	62±19	62±18	0.766
HbA1c (%)	5.8 [5.5–6.2]	5.8 [5.5–6.2]	5.8 [5.5–6.2]	0.617	5.8 [5.4–6.2]	5.8 [5.5–6.2]	0.541

Values are n (%), mean±SD, or median [inter-quartile range]. CAD, coronary artery disease; HADS, Hospital Anxiety and Depression Scale; CRP, C-reactive protein; BNP, B-type natriuretic peptide; eGFR, estimated glomerular filtration rate.

### The relationship between sleep disturbances and psychological disturbances

**Fig 2:**
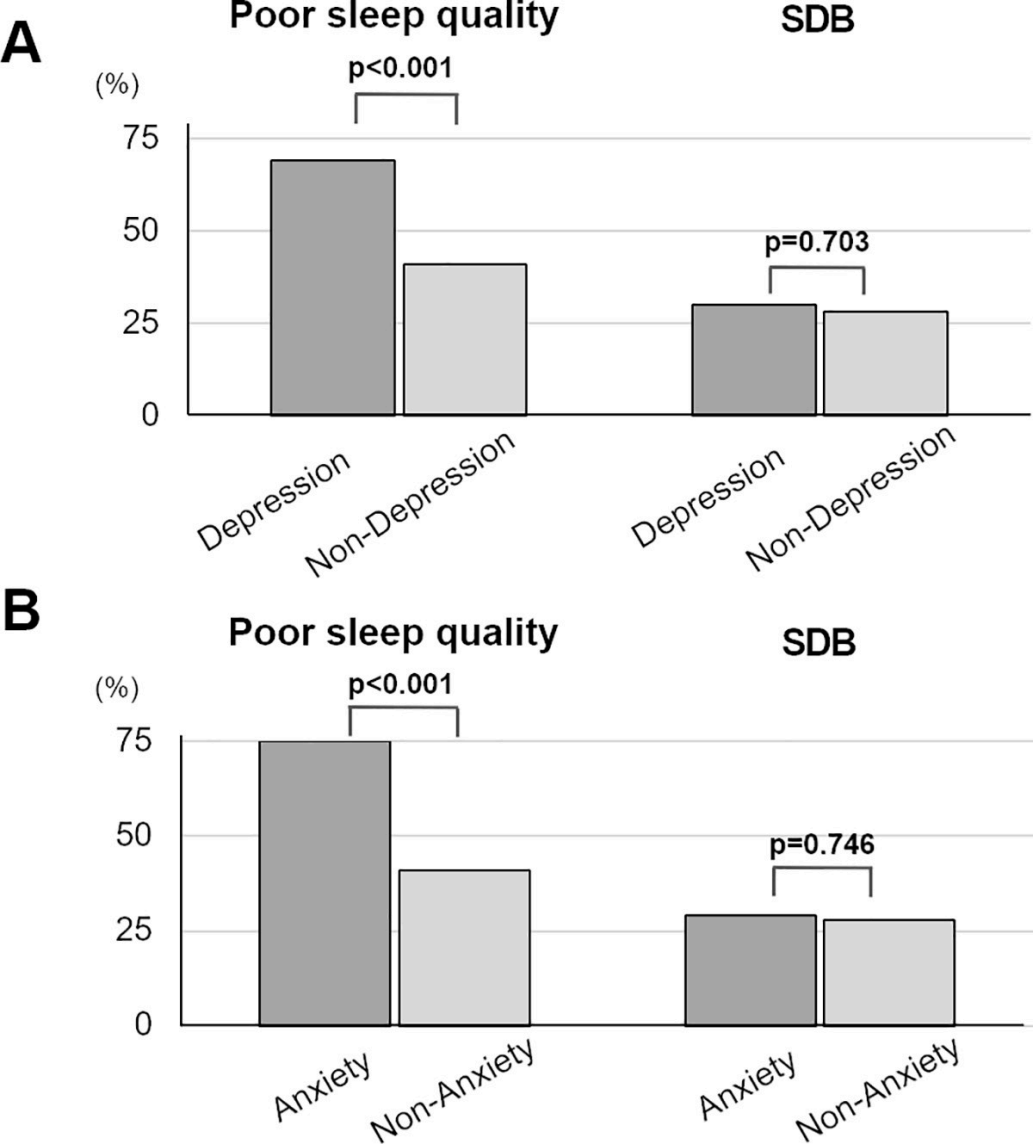
Comparison of poor sleep quality and SDB between patients with and those without psychological disturbances. (A) Depression. (B) Anxiety. SDB, sleep-disordered breathing.

We then analyzed the relationship between psychological disturbances and sleep disturbances. Six hundred two patients (46.5%) were categorized as having a poor sleep quality (PSQI>5), and 369 patients (28.5%) had SDB (3% ODI≥15). The patients with depression or anxiety had a higher prevalence of poor sleep quality (Fig 2A and 2B). Conversely, depression and anxiety symptoms were not associated with the prevalence of SDB (Fig 2A and 2B). The multivariate binary logistic regression analysis for determining the independent determinants of depression or anxiety have been shown in Table 2. Depression and anxiety were independently associated with poor sleep quality (OR: 3.09, 95% CI: 2.19–4.36, OR: 3.93, 95% CI: 2.71–5.69, respectively), while SDB was not (OR: 0.93, 95% CI: 0.64–1.36 and OR: 1.11, 95% CI: 0.74–1.66, respectively). Gender and old age were not statistically significant determinants of depression after adjustment for covariates. The prevalence of HF and dyslipidemia was also a determinant of depression, but was not found to be significant for anxiety.

**Table 2:** Multiple regression analysis of determinants of depression and anxiety.

Variables	Depression		Anxiety	
	OR (95% CI)	P value	OR (95% CI)	P value
Poor sleep quality	3.09 (2.19–4.36)	< 0.001	3.93 (2.71–5.69)	< 0.001
SDB	0.93 (0.64–1.36)	0.709	1.11 (0.74–1.66)	0.605
Age	1.02 (1.00–1.03)	0.063	1.00 (0.98–1.02)	0.939
Gender	1.11 (0.75–1.65)	0.596	1.17 (0.77–1.77)	0.458
Obesity (BMI≥25 kg/m^2^)	1.05 (0.72–1.53)	0.798	0.89 (0.60–1.31)	0.541
Living alone	1.03 (0.66–1.60)	0.914	0.94 (0.58–1.50)	0.781
Medical history				
Coronary artery disease	0.96 (0.63–1.46)	0.851	0.77 (0.49–1.21)	0.249
Heart Failure	1.58 (1.04–2.40)	0.034	1.27 (0.81–2.00)	0.306
Atrial fibrillation	0.90 (0.62–1.31)	0.571	1.04 (0.71–1.53)	0.846
Hypertension	0.76 (0.52–1.09)	0.138	1.51 (1.02–2.25)	0.042
Diabetes mellitus	1.22 (0.81–1.85)	0.340	0.81 (0.50–1.30)	0.376
Dyslipidemia	1.53 (1.06–2.21)	0.024	1.13 (0.76–1.66)	0.548
Laboratory data				
CRP	0.93 (0.76–1.14)	0.496	1.00 (0.82–1.23)	0.976
Albumin	0.91 (0.60–1.38)	0.644	0.88 (0.57–1.36)	0.561
eGFR	1.00 (0.99–1.01)	0.953	1.01 (1.00–1.02)	0.031

OR, odds ratio; CI, confidence interval; SDB, sleep disordered breathing; BMI, body mass index; CRP, C-reactive protein; eGFR, estimated glomerular filtration rate.

**Fig 3:**
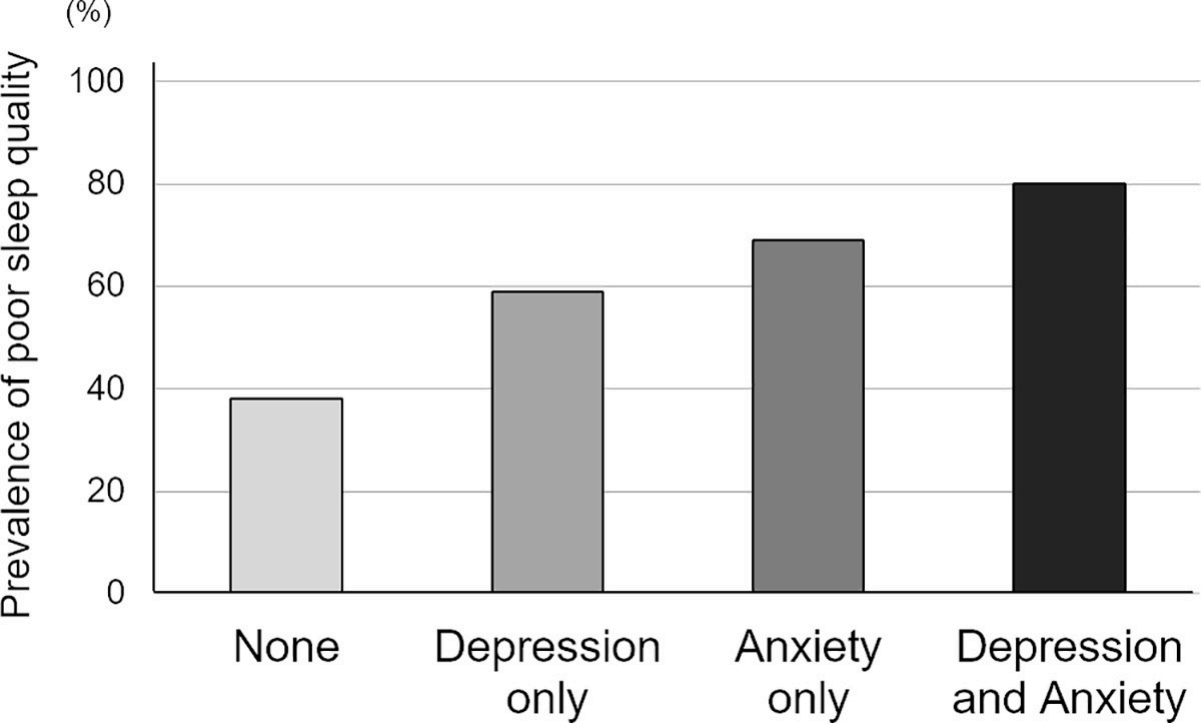
The frequency of poor sleep quality in patients without psychological disturbances (none) and those with depression alone, anxiety alone, and both.

Based on the presence of anxiety and depression, we then created another four groups; there were 949 patients (73.3%) who had neither depression nor anxiety (none), 124 (9.6%) who had depression alone, 101 (7.8%) who had anxiety alone, and 120 (9.3%) who had both. The prevalence of poor sleep quality was 38.3%, 58.9%, 69.3%, and 80.0%, respectively (Fig 3). The adjusted odds ratio of having poor sleep quality was 2.56-fold higher in patients with depression alone (OR, 2.56; 95% CI, 1.64–4.01) and 3.83-fold higher in patients with anxiety alone (OR, 3.83; 95% CI, 2.29–6.41). Concurrently, the adjusted odds ratio was observed to be 4.96-fold higher in patients who had both depression and anxiety (OR, 4.96; 95% CI, 3.00–8.20). On the other hand, there were no significant associations between SDB and psychological disturbances (S1 Table).

## Discussion

### Key findings

In this study, we revealed that 1) approximately one-fifth of patients with CVDs had depression and/or anxiety; 2) patients with depression were more likely to be female and of older age, and had a higher prevalence of HF; and 3) depression and anxiety were strongly associated with poor sleep quality, but not with SDB.

### Interpretation of results

The strength of our study is that our data are based on the consecutive data regarding sleep disturbances (i.e. SDB and poor sleep quality) using pulse oximetry as well as well-validated self-report scales. Interestingly, we found no significant association between SDB and depression or anxiety prevalence, which is inconsistent with the findings from community-based cohorts as well as randomized controlled trials [4–7]. In contrast, poor sleep quality was a remarkable determinant of depression and anxiety, which is concordant with the results from community-based cohorts [8, 9]. From a clinical point of view, clinicians should be aware of their underrecognized coprevalence [20]; poor sleep quality and psychological disturbances might create a vicious cycle worsening each other [21], in addition to being risk factors as well as poor prognostic factors for various CVDs [22–27].

Based on the accumulating evidence that anxiety is independently associated with increased mortality in CVDs, independent from depression [23, 24], screening for anxiety is recommended in addition to that for depression [11, 28]. Depression and anxiety frequently coexist, and in the present study, 54.3% of patients with anxiety had depression symptoms, and 49.2% of patients with depression had anxiety symptoms. Comorbid depression and anxiety were associated with a greater treatment resistance [29, 30], compared with either condition alone. In the treatment-resistant patients, the pharmacological and/or non-pharmacological approaches (e.g. selective serotonin reuptake inhibitors, cognitive behavioral therapy, exercise) [10, 28, 31] need to be tailored to the individual patient's profile by a multidisciplinary team, consisting of cardiologists, psychiatrists, and nurses. Whether health interventions focusing on poor sleep quality (e.g. cognitive therapy or pharmacotherapy) improve the psychological disturbances, particularly among patients with comorbid depression and anxiety in whom poor sleep quality was more prevalent (80.0%), needs to be better understood in the future.

Much of the existing research on psychological disturbances in CVDs has focused on a single specific CVD (e.g. HF, CAD, AF) [22–24, 31–33], and the prevalence of psychological disturbances varied according to the type of CVD and the diagnostic methods. In this large-size sample of patients with a broad range of cardiovascular conditions, we revealed a high prevalence of depression and anxiety in inpatients with various CVDs, particularly in those with a medical history of HF, suggesting that more patients with CVDs could benefit from the early and accurate identification of depression or anxiety. One of the barriers for psychological screening in the cardiology setting is that the effective ways for their assessment remain to be fully elucidated. We used a brief, user-friendly questionnaire, the HADS, which is validated and reliable for the evaluation of depression and anxiety in medically ill hospitalized patients [13, 14]; HADS can be administered to a patient in less than 5 minutes and scored in approximately 1 minute. In addition to the integration of screening in the cardiology setting, overcoming other barriers (lack of cardiologists’ mental health expertise as well as that of downstream support from specialized mental health clinicians) will be needed for the comprehensive management of psychological disturbances.

### Limitations

Several limitations should be noted. First, the nature of our study design (i.e. cross-sectional study) limited the ability to demonstrate any cause-effect relationships between poor sleep quality and psychological disturbances in patients hospitalized for CVDs. Second, several methodological issues need to be mentioned, such as the lack of additional diagnostic assessments with structured instruments or clinical interviews, which generally need to be conducted by mental healthcare providers. Third, the long-term effect of screening and management of psychological disturbances remains unknown. In addition to their adverse association with the prognosis, depression and anxiety could influence the quality of life and treatment adherence (i.e. taking medications as prescribed, participating in cardiac rehabilitation, cooperating with follow-up care, and promoting modifications in standard risk factors) [11, 28, 31]. Whether early identification and interventions for psychological disturbances might improve the prognosis, patient-centered outcomes (quality of life, functional capacity), and treatment adherence, will need to be evaluated comprehensively in the future.

## Conclusions

We found that depression or anxiety were prevalent and independently associated with poor sleep quality, but not with SDB in inpatients with a broad range of CVDs.

## Supporting information

S1 Table Multiple regression analysis of parameters that determine poor sleep quality and sleep disordered breathing. (DOCX)

## References

[1] Disease GBD, Injury I, Prevalence C. Global, regional, and national incidence, prevalence, and years lived with disability for 310 diseases and injuries, 1990–2015: a systematic analysis for the Global Burden of Disease Study 2015. Lancet. 2016;388(10053):1545–602. 10.1016/S0140-6736(16)31678-6 27733282 PMC5055577

[2] Kessler RC, Petukhova M, Sampson NA, Zaslavsky AM, Wittchen HU. Twelve-month and lifetime prevalence and lifetime morbid risk of anxiety and mood disorders in the United States. International journal of methods in psychiatric research. 2012;21(3):169–84. 10.1002/mpr.1359 22865617 PMC4005415

[3] Javaheri S, Barbe F, Campos-Rodriguez F, Dempsey JA, Khayat R, Javaheri S, et al Sleep Apnea: Types, Mechanisms, and Clinical Cardiovascular Consequences. J Am Coll Cardiol. 2017;69(7):841–58. 10.1016/j.jacc.2016.11.069 28209226 PMC5393905

[4] Peppard PE, Szklo-Coxe M, Hla KM, Young T. Longitudinal association of sleep-related breathing disorder and depression. Arch Intern Med. 2006;166(16):1709–15. 10.1001/archinte.166.16.1709 . 16983048

[5] Wheaton AG, Perry GS, Chapman DP, Croft JB. Sleep disordered breathing and depression among U.S. adults: National Health and Nutrition Examination Survey, 2005–2008. Sleep. 2012;35(4):461–7. 10.5665/sleep.1724 22467983 PMC3296787

[6] Alcantara C, Biggs ML, Davidson KW, Delaney JA, Jackson CL, Zee PC, et al Sleep Disturbances and Depression in the Multi-Ethnic Study of Atherosclerosis. Sleep. 2016;39(4):915–25. 10.5665/sleep.5654 26715223 PMC4791625

[7] Campos-Rodriguez F, Queipo-Corona C, Carmona-Bernal C, Jurado-Gamez B, Cordero-Guevara J, Reyes-Nunez N, et al Continuous Positive Airway Pressure Improves Quality of Life in Women with Obstructive Sleep Apnea. A Randomized Controlled Trial. American journal of respiratory and critical care medicine. 2016;194(10):1286–94. 10.1164/rccm.201602-0265OC . 27181196

[8] Jaussent I, Bouyer J, Ancelin ML, Akbaraly T, Peres K, Ritchie K, et al Insomnia and daytime sleepiness are risk factors for depressive symptoms in the elderly. Sleep. 2011;34(8):1103–10. 10.5665/SLEEP.1170 21804672 PMC3138165

[9] Neckelmann D, Mykletun A, Dahl AA. Chronic insomnia as a risk factor for developing anxiety and depression. Sleep. 2007;30(7):873–80. 10.1093/sleep/30.7.873 17682658 PMC1978360

[10] Lichtman JH, Bigger JT, Jr., Blumenthal JA, Frasure-Smith N, Kaufmann PG, Lespesrance F, et al Depression and coronary heart disease: recommendations for screening, referral, and treatment: a science advisory from the American Heart Association Prevention Committee of the Council on Cardiovascular Nursing, Council on Clinical Cardiology, Council on Epidemiology and Prevention, and Interdisciplinary Council on Quality of Care and Outcomes Research: endorsed by the American Psychiatric Association. Circulation. 2008;118(17):1768–75. 10.1161/CIRCULATIONAHA.108.190769 . 18824640

[11] Piepoli MF, Hoes AW, Agewall S, Albus C, Brotons C, Catapano AL, et al 2016 European Guidelines on cardiovascular disease prevention in clinical practice: The Sixth Joint Task Force of the European Society of Cardiology and Other Societies on Cardiovascular Disease Prevention in Clinical Practice (constituted by representatives of 10 societies and by invited experts)Developed with the special contribution of the European Association for Cardiovascular Prevention & Rehabilitation (EACPR). European heart journal. 2016;37(29):2315–81. 10.1093/eurheartj/ehw106 27222591 PMC4986030

[12] Feinstein RE, Blumenfield M, Orlowski B, Frishman WH, Ovanessian S. A national survey of cardiovascular physicians' beliefs and clinical care practices when diagnosing and treating depression in patients with cardiovascular disease. Cardiol Rev. 2006;14(4):164–9. Epub 2006/06/22. 10.1097/01.crd.0000200977.41695.43 . 16788327

[13] Zigmond AS, Snaith RP. The hospital anxiety and depression scale. Acta Psychiatr Scand. 1983;67(6):361–70. 10.1111/j.1600-0447.1983.tb09716.x . 6880820

[14] Bjelland I, Dahl AA, Haug TT, Neckelmann D. The validity of the Hospital Anxiety and Depression Scale. An updated literature review. J Psychosom Res. 2002;52(2):69–77. 10.1016/s0022-3999(01)00296-3 . 11832252

[15] Kimura T, Kohno T, Nakajima K, Kashimura S, Katsumata Y, Nishiyama T, et al Effect of Nocturnal Intermittent Hypoxia on Left Atrial Appendage Flow Velocity in Atrial Fibrillation. Can J Cardiol. 2015;31(7):846–52. 10.1016/j.cjca.2014.12.032 . 25953253

[16] Muraki I, Tanigawa T, Yamagishi K, Sakurai S, Ohira T, Imano H, et al Nocturnal intermittent hypoxia and the development of type 2 diabetes: the Circulatory Risk in Communities Study (CIRCS). Diabetologia. 2010;53(3):481–8. 10.1007/s00125-009-1616-0 . 19946661

[17] Tanigawa T, Tachibana N, Yamagishi K, Muraki I, Umesawa M, Shimamoto T, et al Usual alcohol consumption and arterial oxygen desaturation during sleep. JAMA. 2004;292(8):923–5. 10.1001/jama.292.8.923-b . 15328323

[18] Doi Y, Minowa M, Uchiyama M, Okawa M, Kim K, Shibui K, et al Psychometric assessment of subjective sleep quality using the Japanese version of the Pittsburgh Sleep Quality Index (PSQI-J) in psychiatric disordered and control subjects. Psychiatry Res. 2000;97(2–3):165–72. 10.1016/s0165-1781(00)00232-8 . 11166088

[19] Buysse DJ, Reynolds CF, 3rd, Monk TH, Berman SR, Kupfer DJ. The Pittsburgh Sleep Quality Index: a new instrument for psychiatric practice and research. Psychiatry Res. 1989;28(2):193–213. 10.1016/0165-1781(89)90047-4 . 2748771

[20] Matsuda R, Kohno T, Kohsaka S, Fukuoka R, Maekawa Y, Sano M, et al The prevalence of poor sleep quality and its association with depression and anxiety scores in patients admitted for cardiovascular disease: A cross-sectional designed study. International journal of cardiology. 2017;228:977–82. 10.1016/j.ijcard.2016.11.091 . 27915216

[21] Sivertsen B, Salo P, Mykletun A, Hysing M, Pallesen S, Krokstad S, et al The bidirectional association between depression and insomnia: the HUNT study. Psychosom Med. 2012;74(7):758–65. 10.1097/PSY.0b013e3182648619 . 22879427

[22] Jiang W, Kuchibhatla M, Cuffe MS, Christopher EJ, Alexander JD, Clary GL, et al Prognostic value of anxiety and depression in patients with chronic heart failure. Circulation. 2004;110(22):3452–6. Epub 2004/11/24. 10.1161/01.CIR.0000148138.25157.F9 . 15557372

[23] Watkins LL, Koch GG, Sherwood A, Blumenthal JA, Davidson JR, O'Connor C, et al Association of anxiety and depression with all-cause mortality in individuals with coronary heart disease. J Am Heart Assoc. 2013;2(2):e000068 Epub 2013/03/30. 10.1161/JAHA.112.000068 23537805 PMC3647264

[24] Baumgartner C, Fan D, Fang MC, Singer DE, Witt DM, Schmelzer JR, et al Anxiety, Depression, and Adverse Clinical Outcomes in Patients With Atrial Fibrillation Starting Warfarin: Cardiovascular Research Network WAVE Study. J Am Heart Assoc. 2018;7(8). 10.1161/JAHA.117.007814 29656278 PMC6015441

[25] Laugsand LE, Strand LB, Platou C, Vatten LJ, Janszky I. Insomnia and the risk of incident heart failure: a population study. European heart journal. 2014;35(21):1382–93. 10.1093/eurheartj/eht019 . 23462728

[26] Laugsand LE, Vatten LJ, Platou C, Janszky I. Insomnia and the risk of acute myocardial infarction: a population study. Circulation. 2011;124(19):2073–81. Epub 2011/10/26. 10.1161/CIRCULATIONAHA.111.025858 . 22025601

[27] Kanno Y, Yoshihisa A, Watanabe S, Takiguchi M, Yokokawa T, Sato A, et al Prognostic Significance of Insomnia in Heart Failure. Circulation journal: official journal of the Japanese Circulation Society. 2016;80(7):1571–7. 10.1253/circj.CJ-16-0205 . 27194467

[28] Jha MK, Qamar A, Vaduganathan M, Charney DS, Murrough JW. Screening and Management of Depression in Patients With Cardiovascular Disease: JACC State-of-the-Art Review. J Am Coll Cardiol. 2019;73(14):1827–45. 10.1016/j.jacc.2019.01.041 . 30975301 PMC7871437

[29] Fava M, Rush AJ, Alpert JE, Balasubramani GK, Wisniewski SR, Carmin CN, et al Difference in treatment outcome in outpatients with anxious versus nonanxious depression: a STAR*D report. Am J Psychiatry. 2008;165(3):342–51. 10.1176/appi.ajp.2007.06111868 . 18172020

[30] Stein DJ, Baldwin DS, Baldinetti F, Mandel F. Efficacy of pregabalin in depressive symptoms associated with generalized anxiety disorder: a pooled analysis of 6 studies. Eur Neuropsychopharmacol. 2008;18(6):422–30. 10.1016/j.euroneuro.2008.01.004 . 18359203

[31] Vaccarino V, Badimon L, Bremner JD, Cenko E, Cubedo J, Dorobantu M, et al Depression and coronary heart disease: 2018 ESC position paper of the working group of coronary pathophysiology and microcirculation developed under the auspices of the ESC Committee for Practice Guidelines. European heart journal. 2019 10.1093/eurheartj/ehz413 . 31209462

[32] Rutledge T, Reis VA, Linke SE, Greenberg BH, Mills PJ. Depression in heart failure a meta-analytic review of prevalence, intervention effects, and associations with clinical outcomes. J Am Coll Cardiol. 2006;48(8):1527–37. 10.1016/j.jacc.2006.06.055 . 17045884

[33] Roest AM, Martens EJ, de Jonge P, Denollet J. Anxiety and risk of incident coronary heart disease: a meta-analysis. J Am Coll Cardiol. 2010;56(1):38–46. 10.1016/j.jacc.2010.03.034 . 20620715

