# Supplementary material for: Psychological disturbances and their association with sleep disturbances in patients admitted for cardiovascular diseases
Source: PLoS One. 2021 Jan 4;16(1):e0244484. doi: 10.1371/journal.pone.0244484 (PMC7781384; doi:10.1371/journal.pone.0244484)
Supplement: S1 Table — (DOCX) [file pone.0244484.s001.docx]

S1 Table. Multiple regression analysis of parameters that determine poor sleep quality and sleep disordered breathing

| Variables | Poor sleep quality | | SDB | |
| --- | --- | --- | --- | --- |
|  | OR (95% CI) | P value | OR (95% CI) | P value |
| None | reference |  | reference |  |
| Depression only | 2.56 (1.64-4.01) | <0.001 | 0.83 (0.45-1.54) | 0.562 |
| Anxiety only | 3.83 (2.29-6.41) | <0.001 | 0.70 (0.41-1.18) | 0.182 |
| Depression and anxiety | 4.96 (3.00-8.20) | <0.001 | 1.25 (0.76-2.08) | 0.382 |

OR, odds ratio; CI, confidence interval; SDB, sleep disordered breathing.
